# Rapid Exonuclease‐Based *De Novo* Sequencing Strategy for Long Oligonucleotides via Liquid‐Phase Ion Trap Coupled With Mass Spectrometry

**DOI:** 10.1002/advs.77313

**Published:** 2026-08-20

**Authors:** Song Lu, Jie Hong, Xinyan Fu, Di Wang, Yunhua Gao, Wei Xu

**Affiliations:** ^1^ Center for Advanced Measurement Science National Institute of Metrology Beijing China; ^2^ Kunshan Nier Precision Instrumentation Inc. Kunshan China; ^3^ School of Medical Technology Beijing Institute of Technology Beijing China

**Keywords:** chemistry, computational biology, de novo synthesis, DNA sequencing, electrospray ionization, exonuclease, ion trap, mass spectrometry, oligonucleotide

## Abstract

The sequencing of synthetic oligonucleotides is fundamental to life sciences and therapeutics, yet current analytical approaches present inherent technical drawbacks of varying degrees. Hybridization‐based techniques cannot sequence the oligonucleotide primers they rely on, while the accuracy of mass spectrometry (MS)‐based techniques significantly diminishes as fragment length increases. Here, we introduce a liquid‐phase ion trap (LPIT) coupled with electrospray ionization (ESI)‐MS as a solution for rapid, high‐fidelity oligonucleotide sequencing. The LPIT utilizes a strong electric field to efficiently dissociate confounding metal cation adducts prior to MS analysis, resulting in cleaner spectra and enhanced signal intensity. This “desalting‐first” approach enables direct, rapid oligonucleotide analysis, achieving mass measurement accuracy comparable to benchmark liquid chromatography‐ESI‐MS (LC‐ESI‐MS) platforms. By integrating this platform with an exonuclease digestion workflow, we achieved full sequence coverage of oligonucleotides in under 30 min, a dramatic improvement in speed over existing methods. This work establishes LPIT‐ESI‐MS as a powerful platform for the direct and rapid sequencing of long oligonucleotides, addressing a critical unmet need in biotechnology and drug development.

## Graphical abstract

A liquid‐phase ion trap (LPIT) coupled with ESI‐MS enables rapid, high‐fidelity de novo sequencing of long oligonucleotides in ∼30 min. By applying an electric field to dissociate metal adducts in solution, LPIT delivers clean spectra, superior mass accuracy, and full sequence coverage, surpassing MALDI‐TOF and LC‐MS. This salt‐tolerant, reagent‐free platform addresses a bottleneck in oligonucleotide therapeutics and diagnostics.

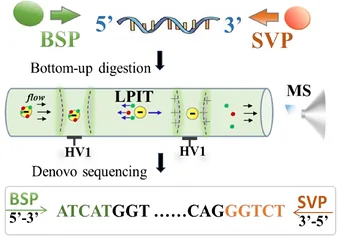

## 1 Introduction

Oligonucleotides are nucleic acid fragments that have become indispensable tools in therapeutic drug development and molecular diagnostics [1, 2, 3, 4, 5, 6]. The clinical safety profiles and target recognition specificity of oligonucleotides are primarily governed by sequence composition [7, 8]. However, current sequencing platforms are poorly suited for short‐stranded oligonucleotides [5, 9, 10, 11]. Gold‐standard methods like Sanger and next‐generation sequencing (NGS) suffer from reduced signal intensity, dye artifacts at the termini, and amplification biases with short fragments, while long‐read technologies are not applicable for fragments shorter than ∼0.5 kb [12, 13]. An alternative approach involves cloning the target fragment into a vector, followed by sequencing, which can provide more accurate results [14]. However, this method requires the design of new primers, molecular cloning, and bacterial cultivation, making it time‐consuming, labor‐intensive, and costly. Besides ultra‐short oligonucleotides like primers, the *de novo* sequencing of larger oligonucleotides, such as human tRNA (73–95 nucleotides) or synthetic aptamer RNA, is critical for revealing complete structural and biological function without prior sequence information [15, 16, 17]. Consequently, there is an urgent demand for rapid and robust methods to sequence oligonucleotides within the 0–100 nt length range. In this study, short oligonucleotides are defined as sequences with a length of less than 30 nucleotides (nt), whereas long oligonucleotides are those longer than 30 nt.

Mass spectrometry (MS) is a natural candidate for this challenge, offering high‐resolution molecular weight determination [9, 18, 19, 20, 21]. Matrix‐Assisted Laser Desorption/Ionization Time‐of‐Flight Mass Spectrometry (MALDI‐TOF) is a powerful technique for sequencing small oligonucleotides, particularly when employing a bottom‐up approach. Controlled exonuclease digestion or acid hydrolysis is routinely applied in this workflow to yield stepwise ladder products. Subsequent MALDI‐TOF analysis enables the identification of individual nucleotides from the mass discrepancies between neighboring fragments (n and n−1) [22, 23]. While this classical method is well‐suited for short oligonucleotides (e.g., <30 nucleotides), Electrospray Ionization Mass Spectrometry (ESI‐MS) has emerged as a dominant tool for oligonucleotide sequencing [24, 25, 26, 27, 28, 29, 30, 31, 32, 33], with two core pretreatment workflows widely implemented: top‐down and bottom‐up. Prior work has documented full sequence coverage for 50–100 nt oligonucleotides; nonetheless, conventional top‐down tandem mass spectrometry (MS/MS) restricts reliable *de novo* sequencing to fragments below 50 nt [22, 34]. When paired with auxiliary separation techniques such as online/offline LC buffer exchange and ion mobility spectrometry [24, 25, 27], longer oligonucleotides can be profiled with enhanced sequencing capability and mass accuracy (Table 1).

**TABLE 1:** Comparison of representative sequencing techniques.

Representative sequencing techniques	Turnaround time	Sequence coverage	Length of oligonucleotides
Sanger sequencing	> 1 day [48, 49, 50, 51]	100%	N.A.
High‐throughput sequencing platforms	several hours [52, 53, 54]	100%	N.A.
Top‐down HPLC‐MS	10‐50 mins [25, 26, 27]	65%–97%	0‐150 nt
Bottom‐up HPLC‐MS	> 2 h [28, 29, 30, 31]	70%–100%	0‐37 nt
LPIT‐ESI‐MS (this work)	< 30 mins	100%	0‐150 nt

Alternatively, bottom‐up ESI‐MS leverages enzymatic digestion of larger RNA molecules, including sgRNA, tRNA, or mRNA, to achieve high or even full sequence coverage [28, 29, 30, 31, 32, 33]. By analyzing shorter digestion fragments or incorporating ion‐pairing strategies within the LC system, this method can provide a more comprehensive analysis of complex nucleic acid structures. Notably, a 2D hydrophobic RNA end‐labeling strategy integrated with an anchor‐based algorithm for mass spectrometry‐based sequencing (2D‐HELS‐AA MS Seq) and a mass spectrometry ladder complementation sequencing approach (MLC‐Seq) have been developed specifically for the *de novo* MS sequencing of full‐length tRNAs [28, 31]. Both techniques are adept at unveiling comprehensive information regarding the target tRNA by leveraging a “ladder” of oligonucleotide fragments in conjunction with a meticulously designed anchor‐based algorithm.

The core issue for analyzing nucleic acids is the polyanionic phosphodiester backbone, which has a strong affinity for metal cations [35]. Even trace amounts of salt lead to a wide array of adduct species, which fractures the ion signal, suppresses sensitivity, and makes accurate mass assignment nearly impossible [36]. Conventional desalting approaches such as precipitation and solid‐phase extraction work well for short nucleic acid fragments, yet exhibit insufficient desalting efficiency toward longer nucleic acids [37]. Precipitation may suffer from sample loss. More importantly, larger nucleic acids, which possess a pronounced propensity for cation adduction, can be susceptible to even contamination from ions leaching from container surfaces during the analysis process. A range of online buffer‐exchange techniques, including microflow‐ and nanoflow‐based approaches, have been recently designed for effective desalting and high mass accuracy with longer oligonucleotides [25, 38]. Ion‐pairing reagents (IPRs) represent the classical desalting approach adopted in reversed‐phase LC‐MS (RP‐LC‐MS) [37, 39, 40, 41, 42], while hydrophilic interaction liquid chromatography coupled with ESI‐MS (HILIC‐ESI‐MS) serves as an alternative ion‐pair‐free strategy [30, 43]. Both methods achieve efficient elimination of nucleic acid cation adducts and enhance overall oligonucleotide sequencing performance. Collectively, these established LC‐MS‐dependent pipelines highlight the demand for alternative ESI‐MS platforms enabling direct, rapid characterization of intact large oligonucleotide fragments to further elevate overall sequencing coverage and throughput.

To directly resolve the cation adduct interference that impairs oligonucleotide mass analysis, we adapted our recently developed liquid‐phase ion trap (LPIT) system [44, 45, 46]. It was found that applying a negative DC electric field within the LPIT effectively dissociates metal cation adducts from oligonucleotides while simultaneously enriching the purified analyte. The strong electric field applied between the two HV metal connectors within the LPIT region can exert opposite forces on the polyanionic oligonucleotides and the metal cations, thus pulling them apart within the liquid flow. This approach circumvents the issues of adduct formation that plague conventional MS and eliminates the need for instrument‐contaminating IPRs. We demonstrate a complete workflow for the multiplexed analysis and rapid *de novo* sequencing of oligonucleotides up to 53 nt in less than 30 min, demonstrating a powerful new strategy for rapid and high‐fidelity oligonucleotide analysis.

## 2 Result

**FIGURE 1:**
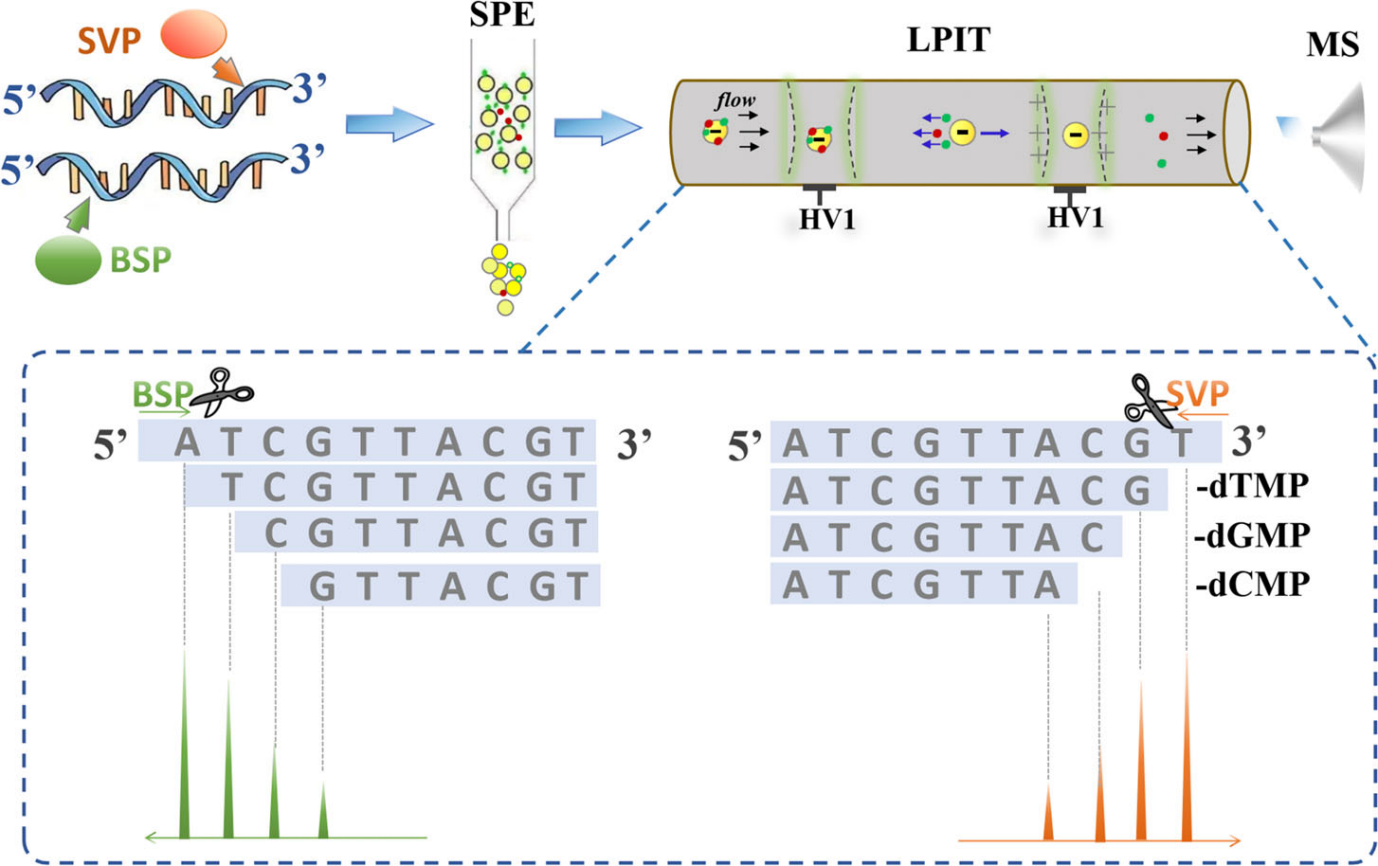
Workflow for oligonucleotide sequencing using the LPIT‐ESI‐MS system. Digested fragment series (differ by one nucleotide base) by exonuclease either from 5’‐3’ or 3’‐5’ are purified and analyzed via LPIT‐ESI‐MS.

For this study, a regulated exonuclease digestion strategy integrated with the newly established LPIT‐ESI‐MS technique was developed for *de novo* sequencing of long oligonucleotides (Figure 1). Through time‐ and concentration‐dependent reaction regulation, exonucleases progressively excise single nucleotides sequentially from either the 5’ or 3’ terminus of the parent oligonucleotide. This process generates a ladder of digested fragments, where adjacent products differ precisely by one nucleotide in length. The obtained mixture of graded‐length oligonucleotide fragments from the two directions was further analyzed by LPIT‐ESI‐MS to realize reliable full‐sequence identification.

Prior to sequencing verification, the analytical performance of LPIT‐ESI‐MS was systematically assessed. Direct infusion tests using individual short oligonucleotides and long oligonucleotide mixtures were first conducted to verify its desalting capacity. Afterward, the serial exonuclease digestion products were measured to accomplish accurate sequence elucidation. In brief, the core principle of the LPIT is the application of an electric field to dissociate positively charged metal cations from the nucleic acid‐metal adducts, where the intense electric field inside the LPIT zone generates opposite forces on polyanionic oligonucleotides and metal cations, enabling their efficient separation in liquid flow (Figure S1). This process exposes the negatively charged phosphate groups on the nucleic acid backbone, which then facilitates the molecule's temporary adsorption and enrichment onto a metal electrode within the trap. Upon removal of the electric field, the now “clean” and concentrated nucleic acid ions are eluted directly into the ESI source for detection over a period of 1.10 min (Figure S2). The primary benefit of this approach is the generation of simplified mass spectra that are free from confounding adducts, leading to a significant enhancement in signal intensity and enabling the determination of highly accurate molecular weights. Sequence determination is achieved through the calculation of mass differences between two adjacent fragments with closely related molecular weights within the sequencing ladder.

**FIGURE 2:**
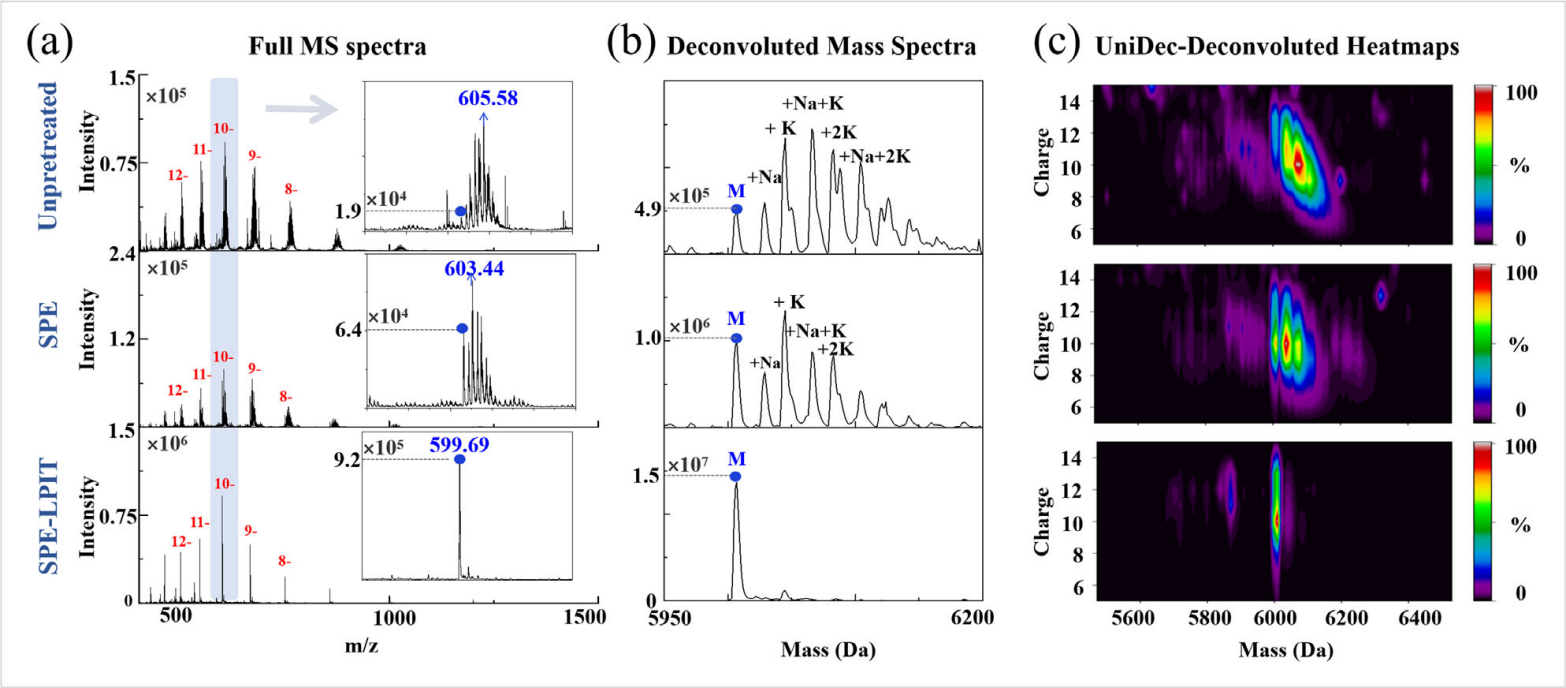
Mass spectra of ssDNA S1 using different treatment techniques. Unpretreated group: Direct infusion of sample S1 into ESI‐MS without pretreatment. SPE group: S1 analyzed by ESI‐MS after solid‐phase extraction. SPE‐LPIT group: S1 determined by LPIT‐ESI‐MS following SPE pretreatment. Figure 2a: full mass spectra; Figure 2b: deconvoluted mass spectra; Figure 2c: UniDec‐deconvoluted heatmaps [47] of mass vs. charge plot.

System performance was first evaluated using a 20 nt single‐stranded DNA template (S1, Table S1) under previously optimized conditions (1.8 × 10^5^ V/m electric field, 2 µL/min flow rate). A continuous flow injection analysis (FIA) mode was utilized for sample injection, and free salt ions remaining in the buffer could promote the re‐adduction of cations to the nucleic acid after dissociation in the LPIT. Therefore, we incorporated a solid‐phase extraction (SPE) step upstream of the LPIT system to reduce the concentration of free salts. The results of this optimization are presented in Figure 2, which compares three conditions: direct injection (unpretreated), SPE pretreatment alone, and the combined SPE‐LPIT approach. The combined SPE‐LPIT method yielded a substantial 46‐fold enhancement in the target signal compared to the untreated sample. In contrast, SPE alone provided only a 3‐fold increase in signal intensity.

Deconvolution of the mass spectra reveals the reason for this dramatic improvement. In both the direct injection and SPE‐only samples, the spectra are dominated by prominent adduct ion peaks, corresponding to various metal ions binding to the nucleic acid at different stoichiometries. The SPE‐LPIT approach, however, markedly reduces the intensity of these adduct peaks, producing a significantly cleaner spectrum. This simplification is visualized in the deconvoluted heatmaps, which demonstrate that the LPIT confines the signal to a much narrower and more accurate mass and charge range. These results confirm a significant improvement in both resolution and signal specificity, which is essential for the precise analysis of target fragments. Furthermore, a two‐stage SPE (SPE+SPE) desalting experiment was also carried out and compared with SPE+LPIT (Figure S3). Results indicate that the SPE procedure alone is insufficient to fully eliminate metal adducts, particularly for oligonucleotides exceeding 30 nucleotides in length. This observation underscores the importance of utilizing LPIT for the effective mitigation of metal adduction, thereby enhancing the overall reliability and accuracy of mass spectrometric analyses.

**FIGURE 3:**
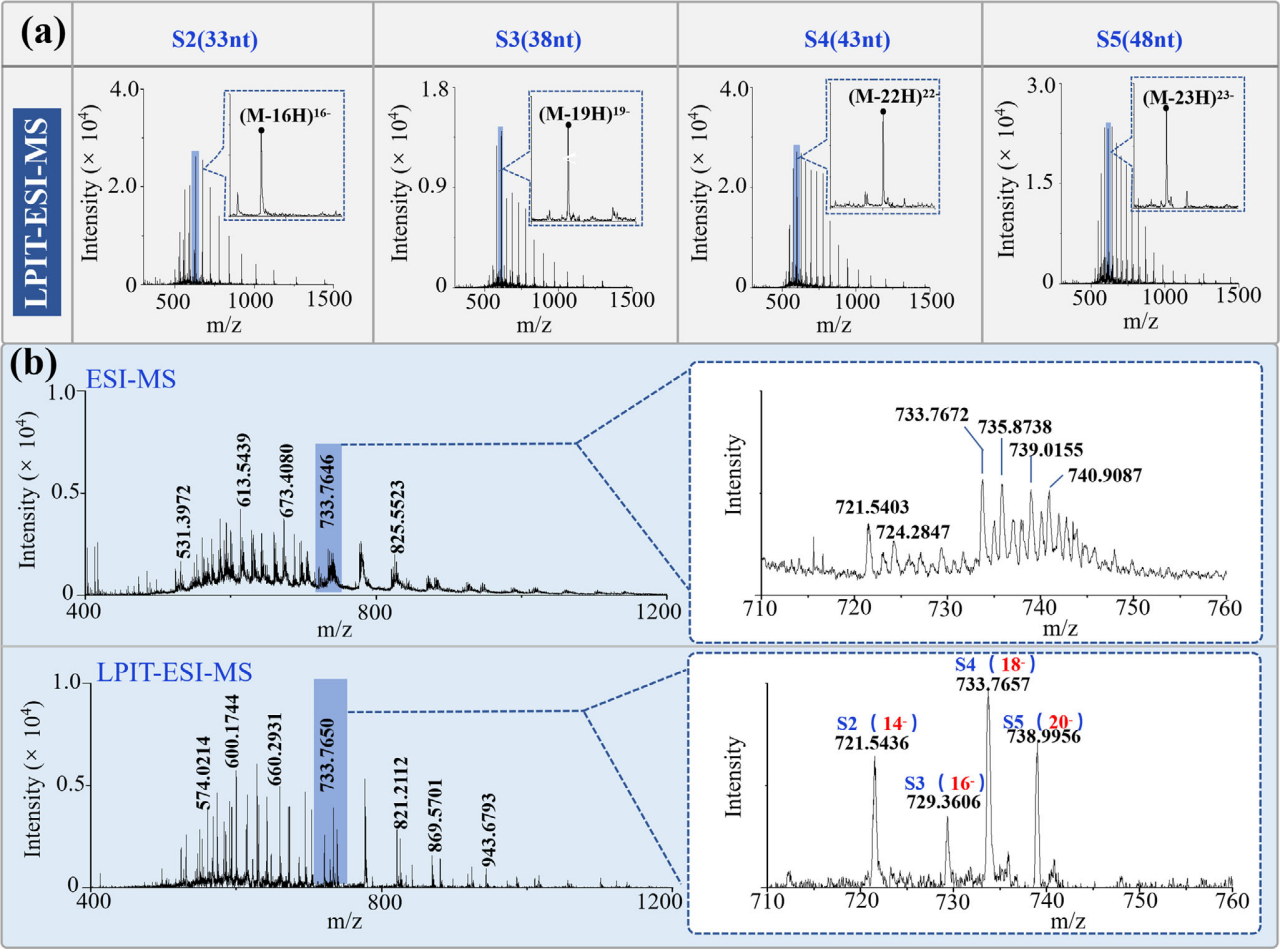
Individual and multiplexed detection of four fragments by ESI‐MS or LPIT‐ESI‐MS. The upper section of panel (a) mass spectrum of each fragment obtained using LPIT‐ESI‐MS. Panel (b) mass spectra of the mixed sample detected under both ESI‐MS and LPIT‐ESI‐MS conditions.

To assess the system's capability for multiplexed analysis, we prepared a mixture of four distinct oligonucleotide fragments ranging from 33 to 48 nt (S2, S3, S4, and S5; Table S1). First, each fragment was analyzed individually using LPIT‐ESI‐MS (Figure 3a). The observed molecular weights showed excellent agreement with their theoretical values, with mass deviations of 9.9, 25.7, 22.7, and 33.8 ppm for S2, S3, S4, and S5, respectively. When the four‐fragment mixture was analyzed, the LPIT‐ESI‐MS system clearly resolved distinct peaks for each component (Figure 3b bottom), and the deconvoluted molecular weights remained highly accurate, validating the method's reliability for complex samples. On the other hand, conventional ESI‐MS failed to detect all four components (Figure 3b top). Salt adducts obscured the primary analyte signals, and only two of the four fragments (S2 and S4) were discernible above the background noise. A similar comparison with MALDI‐TOF MS also highlighted the advantages of our system (Figure S4). When analyzing just a single fragment (S2), MALDI‐TOF produced a mass deviation of 603 ppm (∼60 times higher than our method), and it failed to resolve the components of the mixture. This is attributed to the inherent mass accuracy limitations of MALDI‐TOF and its susceptibility to salt adducts, especially for larger oligonucleotides. In addition, a mixture of three distinct oligonucleotide fragments (S7, S8, S10; Table S1) was analyzed by RP‐LC‐MS. S8 with a length of 33 nt and S7 with a length of 53 nt cannot be identified (Figure S5) under the condition of 15 mM ammonium acetate. We further compared the analytical performance of LPIT‐ESI‐MS and IP‐RP‐LC‐MS using this three‐component oligonucleotide mixture (S7, S8, S10) (Figure S6). LPIT‐ESI‐MS allowed simultaneous deconvolution of all target fragments, while IP‐RP‐LC‐MS required deconvolution of individual fragments separately. Notably, analyses were performed at an analyte concentration of 1 µM for LPIT‐ESI‐MS, in contrast to 10 µM for IP‐RP‐LC‐MS. These results demonstrate that LPIT‐ESI‐MS affords faster analysis of distinct long oligonucleotides without the need for IPRs and with substantially lower sample consumption.

**FIGURE 4:**
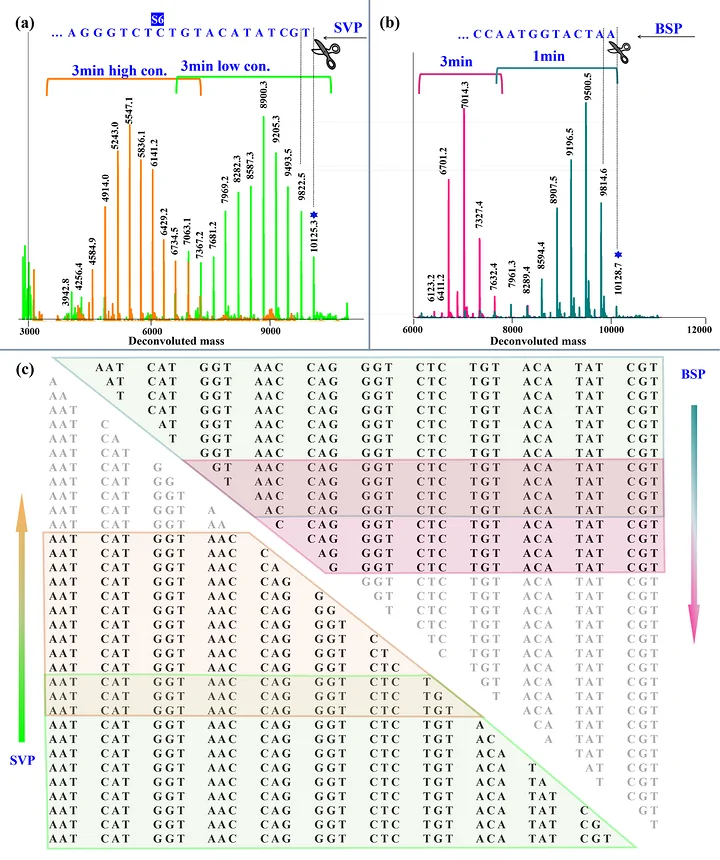
Sequencing results for S6. Figure 4a: deconvoluted mass spectrum for S6 with SVP treatments, low concentration (low con.): 0.6 mU/µL and high concentration (high con.): 1.0 mU/µL. Figure 4b: deconvoluted mass spectrum for S6 with BSP treatments. Figure 4c: digested sequences of each enzyme from two directions with confirmation of complete sequence coverage by the overlapped region.

Finally, we applied the platform to its ultimate challenge: de novo oligonucleotide sequencing. We generated sequencing ladders from two fragments, a 33 nt (S6) and a 53 nt (S7), using bidirectional exonuclease digestion. SVP was employed to digest the fragments from the 3’‐terminus, with varying incubation times, while BSP was utilized for digestion in the 5’‐3’ direction. The resulting digest mixtures were rapidly desalted via SPE and analyzed by LPIT‐ESI‐MS. For the 33 nt fragment, we obtained full, bidirectional sequence coverage (Figure 4). The deconvoluted mass spectra revealed a clean ladder of fragments, with measured molecular weights deviating by less than 1 Da from their theoretical values. The mass differences between consecutive peaks directly corresponded to the expected deoxynucleotide monophosphates (dNMPs), allowing us to read the sequence unambiguously. To further reduce turnaround time, as illustrated in Figure S7, the analytical procedure was optimized by consolidating the digested products from a single direction into one injection. Consequently, two injections with two directions are sufficient to achieve complete sequence coverage. Therefore, the entire process, from digestion to final spectrum, can be completed under 24 min (∼3 min for digestion, 5 min for SPE, followed by 8 min for each combined injection, resulting in a total of 16 min for LPIT‐ESI‐MS), a dramatic reduction in analysis time compared to traditional methods [48, 49, 50, 51].

The method proved equally robust for the longer 53 nt fragment, achieving full sequence coverage within four injections, which can also be reduced to 30 min by combining digested products (Figure S8). LPIT's ability to resolve a complex mixture from a single time point is critical for high‐throughput workflows. As shown in Table S2, a single digest mixture from a 10 min incubation yielded 19 distinct ladder fragments, all of which were detected simultaneously with a mass accuracy of better than 100.6 ppm. Table 1 compares the turnaround time of LPIT‐ESI‐MS with state‐of‐the‐art sequencing techniques. The LPIT‐ESI‐MS platform demonstrates a significantly shorter turnaround time for sequencing compared to Sanger and high‐throughput methods. Crucially, unlike other HPLC‐ESI‐MS techniques, our method operates without IPRs. This IPR‐free workflow prevents instrument contamination and avoids health hazards, positioning it as a robust and accessible solution for future sequencing applications.

## 3 Conclusion

In this work, we have developed a rapid, MS‐based method that overcomes the long‐standing challenges of sequencing oligonucleotides longer than 30 nucleotides. By coupling a bottom‐up exonuclease strategy with our innovative LPIT‐ESI‐MS system, we efficiently eliminate confounding metal adducts. These yield enhanced sensitivity and provide the high mass accuracy required for unambiguous single nucleotide identification, matching the sequencing performance achieved by state‐of‐the‐art LC‐ESI‐MS while overcoming key limitations of conventional MALDI‐MS. The practical result is a dramatic increase in throughput and speed. We achieved full sequence coverage of two large oligonucleotide fragments in a significantly reduced turnaround time and resolved up to 19 ladder fragments in a single run. This represents a significant time‐saving advantage over traditional sequencing methods. As the demand for synthetic oligonucleotides as therapeutics and diagnostics continues to grow, rapid and reliable sequencing platforms like the one demonstrated here will become indispensable, such as for quality control of therapeutic oligonucleotides, the validation of diagnostic probes (e.g., FISH), and the characterization of functional RNAs (e.g., sgRNAs). Given that heavily modified oligonucleotides may impede the catalytic activity of the exonuclease utilized herein during *de novo* sequencing workflows, the integration of LPIT‐ESI‐MS with orthogonal nucleases such as nuclease P1 [55] represents a promising analytical paradigm for *de novo* sequencing of therapeutically relevant siRNA species or tRNAs.

## Author Contributions

**Jie Hong**: conceptualization, methodology, data curation, formal analysis, validation, visualization. **Di Wang**: validation, investigation. **Wei Xu**: conceptualization, methodology, funding acquisition, project administration, resources, writing – review and editing, visualization. **Song Lu**: conceptualization, methodology, data curation, investigation, validation, writing – original draft. **Yunhua Gao**: funding acquisition, validation, supervision, project administration. **Xinyan Fu**: data curation, validation.

## Conflicts of Interest

The authors declare no conflicts of interest.

## Supporting information




**Supporting File**: advs77313‐sup‐0001‐SuppMat.docx.

## Data Availability

The data that support the findings of this study are available on request from the corresponding author. The data are not publicly available due to privacy or ethical restrictions.
