# Supplementary material for: Rapid Exonuclease‐Based De Novo Sequencing Strategy for Long Oligonucleotides via Liquid‐Phase Ion Trap Coupled With Mass Spectrometry
Source: Adv Sci (Weinh). 2026 Aug 20;13(62):e77313. doi: 10.1002/advs.77313 (PMC13491358; doi:10.1002/advs.77313)
Supplement: Supplementary file 1 [file ADVS-13-e77313-s001.docx]

*Supporting Information*

**Rapid Exonuclease-Based De Novo Sequencing Strategy for Long Oligonucleotides via Liquid-Phase Ion Trap coupled with Mass Spectrometry**

Song Lu^1#^, Jie Hong^2#^, Xinyan Fu^3^, Di Wang^1^, Yunhua Gao^1*^ and Wei Xu^3*^

^1^ Center for Advanced Measurement Science, National Institute of Metrology, Beijing, 100029, China

^2^Kunshan Nier Precision Instrumentation Inc., Kunshan, 215316, China

^3^School of Medical Technology, Beijing Institute of Technology, Beijing 100081, China

*Corresponding Author:

| Yunhua Gao  Center for Advanced Measurement Science  National Institute of Metrology  Chaoyang, Beijing, 100029, China  Email: [gaoyh@nim.ac.cn](mailto:gaoyh@nim.ac.cn) | Wei Xu  School of Medical Technology  Beijing Institute of Technology  Haidian, Beijing, 100081, China  Email: weixu@bit.edu.cn |
| --- | --- |

#Equal Contribution

Chemicals and materials

Oligonucleotides listed in Table S1 were custom synthesized by Sangon Biotechnology Co., Ltd. (Shanghai, China). Acetonitrile (ACN) of HPLC grade was sourced from Fisher Chem. Alert Co. (NJ, USA), while deionized water was obtained from Watson (Hong Kong, China). BSP, SVP, ammonium acetate, and sodium acetate were obtained from Sigma‑Aldrich (St. Louis, MO, USA). 10×PBS and triethylamine acetate (TEAA) were supplied by Thermo Fisher Scientific (Waltham, MA, USA). All chemicals were used as received, without any additional purification. Stock solutions of the samples were prepared in water at a concentration of 100 μmol/L and subsequently diluted to the desired concentrations using a running buffer composed of a 1:1 (v/v) mixture of ACN and water before LPIT-ESI-MS. The RP-LC-MS or IP-RPLC-MS samples were diluted to a final concentration of 10 μmol/L before use.

Instrument setup

The experimental setup consists of four main components: a sample injection system, a capillary fluid channel, steel connectors for electrodes, and a mass spectrometer for detection. In the flow injection analysis (FIA) mode, a 0.5 mL syringe filled with the sample is injected into the liquid channel using a syringe pump (Harvard, No. 704500), which also ensures a consistent liquid flow rate for the liquid-phase ion trap. Alternatively, a six-way microvalve (Agilent 1200 series, 2-position/6-port, No. G1162A) can be employed for sample injection. The sample initially passes through an inline MicroFilter kit (Agilent, No. 5067-1582) to filter out microparticles and air bubbles from the liquid flow. The capillary fluid channels used in the experiments are made from PEEKsil capillaries (i.d. 150 μm, o.d. 1/32 inch). The capillary between the MicroFilter and the first stainless steel connector (VICI, No. ZU1C) has a length of 50 cm, and the distance between the first and second metal connectors is 5 cm. A tunable negative high voltage (-HV) is applied to the first connector, provided by a high voltage generator (PRB15R8M1LR2RT2LC1, Wisman Inc., Xianyang, China). The second metal connector is grounded during the experiment. The liquid-phase ion trap is linked to the mass spectrometer (Agilent G6520B Accurate-Mass Q-ToF) via an electrospray ionization (ESI) source, with the default configuration directly connecting to the second connector, unless otherwise noted. The mass spectrometry parameters used during the experiment are as follows: negative ionization mode, ionization voltage of 4000 V, gas temperature set at 250 °C, gas flow rate of 8.0 L/min, fragmentor voltage of 120 V, and a mass range of m/z 300–3000 for data acquisition.

MALDI-TOF mass spectrometry was performed on a Bruker autoflex maX instrument, operating in linear positive ion mode (Ionization Mode LD+). Spectra were recorded with an acceleration voltage of 20 kV, covering a mass range from m/z 3000 to 20,000. Delayed extraction (DE) was employed, with a delay time of 400 ns. The laser energy was adjusted to 30% above the threshold, and a total of 10,000 laser pulses were accumulated for each spectrum.

RP-LC-MS and IP-RP-LC-MS was also performed on Agilent 1200 series HPLC coupled to Agilent G6520B Accurate-Mass Q-ToF. The mass spectrometry parameters were set as described in the previous sections. A Waters XBridge BEC300 column, 3.5 μm, 2.1 × 150 mm was used. For RP-LC-MS, Mobile phase A consisted of 15 mM ammonium acetate (pH = 8.8), and Mobile Phase B consisted of 15 mM ammonium acetate (pH = 8.8) in 40% ACN. The gradient was as follows: 0−15% B from 0 to 25 min, 15-50% B from 25 to 28 min, 50-100% B from 28 to 29min, 100% B from 29 to 31 min, 100-1% B from 31 to 32 min, and 1% B from 32-38 min. The column compartment was kept at 30 °C, and flow rate was 0.2 mL/min. Injection volume was 10 μl. For IP-RP-LC-MS, Mobile phase A consisted of 20 mM triethylamine acetate (TEAA) (pH = 7.0) in 5% ACN., and Mobile Phase B 20 mM triethylamine acetate (TEAA) (pH = 7.0) in 25% ACN. The gradient was as follows: 0−70% B from 0 to 28 min, 70-100% B from 28 to 29 min, 100% B from 29 to 31 min, 100-0% B from 31 to 32 min, and 0% B from 32-40 min. The column compartment was kept at 30 °C, and flow rate was 0.15 mL/min. Injection volume was 10 μl.

Exonuclease digestion

Sodium acetate (20 mM) was employed as the buffer for BSP digestion, while a 0.4×PBS (pH 7.3 ± 0.1) solution was utilized as the buffer for SVP digestion. Each digestion reaction was incubated for various durations, specifically 1, 3, 10, 15, 20, and 30 minutes. The enzyme concentration and reaction time were both optimized to determine the optimal conditions for the digestion process.

For the BSP digestion of S7, 3000 pmol of S7 was incubated with 200 mU of BSP in sodium acetate to a final volume of 100 μL. Similarly, for the SVP digestion of S7, 3000 pmol of S7 was mixed with 300 mU of SVP in 0.4×PBS, also with a total volume of 100 μL. For BSP digestion of S6, 3000 pmol of S6 was combined with 150 mU of BSP in sodium acetate, with a total volume of 100 μL. For SVP digestion of S6 (high concentration), 3000 pmol of S6 was incubated with 100 mU of SVP in 0.4×PBS, with a total volume of 100 μL. For SVP digestion of S6 (low concentration), 3000 pmol of S6 was incubated with 60 mU of SVP in 0.4×PBS, with a total volume of 100 μL.

Supplementary Figure

**Figure S1**. The desalting performances of SPE-MS, SPE-SPE-MS, and SPE-LPIT-MS. Left panel is the spectra of ssDNA S8 with a length of 33 nt. Right panel is the spectra of ssDNA S7 with a length of 53 nt.

**Figure S2**. TIC of ssDNA S1 by LPIT-ESI-MS. The duration of the eluting ions (sample S1) using the LPIT is 1.10 min.

**Figure S3**. The desalting performances of SPE-MS, SPE-SPE-MS, and SPE-LPIT-MS. Left panel is the spectra of ssDNA S8 with a length of 33 nt. Right panel is the spectra of ssDNA S7 with a length of 53 nt.

**Figure S4.** S2 and the mixture of S2,S3,S4,S5 results of MALDI-MS. The bottom figure in the left section shows the LPIT-ESI-MS results of S2. The bottom figure in the right section shows the LPIT-ESI-MS results of the mixture.

**Figure S5**. RP-LC-MS analysis of oligonucleotides mixture (15nt/33nt/53nt). Upper section is the TIC and EIC data of S10 (15 nt). Middle section is the TIC and EIC data of S8 (33 nt). Bottom section is the TIC and EIC data of S7 (53 nt).

**Figure S6**. LPIT-ESI-MS and IP-RP-LC-MS analysis of oligonucleotides mixture (15nt/33nt/53nt). (a) Deconvoluted mass spectra acquired by LPIT-ESI-MS. (b) EIC and corresponding deconvoluted mass spectra obtained via IP-RP-LC-MS.

**Figure S7.** Sequencing results for S6. The left panel displays the deconvoluted mass spectrum of S6 by a single injection with two digested products using SVP. The right panel displays the deconvoluted mass spectrum of S6 by a single injection with two digested products using BSP.

**Figure S8.** Sequencing results and digestion times for S7. The left panel displays the sequencing data with the corresponding molecular weights of the sequencing ladders and the digestion times for SVP treatments. The right panel presents the sequencing results with the molecular weights of the sequencing ladders and the digestion times for BSP treatments.

Supplementary Table

**Table S1.** List of six different lengths of ssDNAs used for desalination effect characterization

| No | Sequence(5’-3’) | Length(nt) | Theoretical | Measured | Difference (ppm) |
| --- | --- | --- | --- | --- | --- |
| S1 | GCC/rU/CAGTCTGCTTCGCACC | 20 | 6007.0 | 6007.1 | 16.7 |
| S2 | ACCAGGTTGCTGTTCTTTATCAGGTTGTTAACT | 33 | 10115.6 | 10115.7 | 9.9 |
| S3 | CCTCTCTAGGGCAGTCGGTGATCGCAGTACGATCGTA | 38 | 11685.6 | 11685.9 | 25.7 |
| S4 | CCTCTCTATGGGCAGTCGGTGATCGCAGTACGATCGTATCTAG | 43 | 13225.6 | 13225.9 | 22.7 |
| S5 | CCTCTCTATGGGCAGTCGGTGATCGCAGTACGATCGTATCTAGCAGAG | 48 | 14799.6 | 14800.1 | 33.8 |
| S6 | AATCATGGTAACCAGGGTCTCTGTACATATCGT | 33 | 10127.7 | 10126.8 | 88.9 |
| S7 | CCTCTCTATGGGCAGTCGGTGATCGCAGTACGATTCTCCACACTTTATACAGG | 53 | 16234.6 | 16235.2 | 37.0 |
| S8 | ACCAGGTTGCTGTTCTTTATCAGGATGTTAACT | 33 | 10124.6 | 10124.4 | 19.8 |
| S9 | ACCAGGTTGCTGTTCTTTATCAGGGTGTTAACT | 33 | 10140.6 | 10140.0 | 59.2 |
| S10 | GAAGGTTTTCCTTCG | 15 | 4574.0 | 4574.0 | 0.0 |

**Table S2.** List of the 19 sequencing ladders of S7 by SVP for the 10-minute incubation time

| No | Sequence(5’-3’) | Length(nt) | Theoretical | Measured | Difference (ppm) |
| --- | --- | --- | --- | --- | --- |
| 1 | CCTCTCTATGGGCAGTCGGTGATCGCAGTACGATTCTCCACAC | 43 | 13130.5 | 13129.9 | 45.7 |
| 2 | CCTCTCTATGGGCAGTCGGTGATCGCAGTACGATTCTCCACA | 42 | 12841.4 | 12842.1 | 54.5 |
| 3 | CCTCTCTATGGGCAGTCGGTGATCGCAGTACGATTCTCCAC | 41 | 12528.2 | 12528.3 | 8.0 |
| 4 | CCTCTCTATGGGCAGTCGGTGATCGCAGTACGATTCTCCA | 40 | 12239.0 | 12238.1 | 73.5 |
| 5 | CCTCTCTATGGGCAGTCGGTGATCGCAGTACGATTCTCC | 39 | 11925.8 | 11926.8 | 83.9 |
| 6 | CCTCTCTATGGGCAGTCGGTGATCGCAGTACGATTCTC | 38 | 11636.6 | 11636.1 | 34.4 |
| 7 | CCTCTCTATGGGCAGTCGGTGATCGCAGTACGATTCT | 37 | 11347.4 | 11346.8 | 52.9 |
| 8 | CCTCTCTATGGGCAGTCGGTGATCGCAGTACGATTC | 36 | 11043.2 | 11042.8 | 36.2 |
| 9 | CCTCTCTATGGGCAGTCGGTGATCGCAGTACGATT | 35 | 10754.0 | 10753.8 | 18.6 |
| 10 | CCTCTCTATGGGCAGTCGGTGATCGCAGTACGAT | 34 | 10449.8 | 10449.7 | 9.6 |
| 11 | CCTCTCTATGGGCAGTCGGTGATCGCAGTACGA | 33 | 10145.6 | 10144.7 | 88.7 |
| 12 | CCTCTCTATGGGCAGTCGGTGATCGCAGTACG | 32 | 9832.4 | 9832.5 | 10.2 |
| 13 | CCTCTCTATGGGCAGTCGGTGATCGCAGTAC | 31 | 9503.2 | 9502.6 | 63.1 |
| 14 | CCTCTCTATGGGCAGTCGGTGATCGCAGTA | 30 | 9214.0 | 9213.5 | 54.3 |
| 15 | CCTCTCTATGGGCAGTCGGTGATCGCAGT | 29 | 8900.8 | 8900.5 | 33.7 |
| 16 | CCTCTCTATGGGCAGTCGGTGATCGCAG | 28 | 8596.6 | 8596.4 | 23.3 |
| 17 | CCTCTCTATGGGCAGTCGGTGATCGCA | 27 | 8267.4 | 8267.4 | 0.0 |
| 18 | CCTCTCTATGGGCAGTCGGTGATCGC | 26 | 7954.2 | 7953.4 | 100.6 |
| 19 | CCTCTCTATGGGCAGTCGGTGATCG | 25 | 7665.0 | 7664.3 | 91.3 |
